# Operando NMR spectroscopic analysis of proton transfer in heterogeneous photocatalytic reactions

**DOI:** 10.1038/ncomms11918

**Published:** 2016-06-17

**Authors:** Xue Lu Wang, Wenqing Liu, Yan-Yan Yu, Yanhong Song, Wen Qi Fang, Daxiu Wei, Xue-Qing Gong, Ye-Feng Yao, Hua Gui Yang

**Affiliations:** 1 Key Laboratory for Ultrafine Materials of Ministry of Education, School of Materials Science and Engineering, East China University of Science and Technology, Shanghai 200237, China; 2 Department of Physics, Shanghai Key Laboratory of Magnetic Resonance, East China Normal University, Shanghai 200062, China; 3 Key Laboratory for Advanced Materials, Centre for Computational Chemistry, Research Institute of Industrial Catalysis, East China University of Science and Technology, Shanghai 200237, China; 4 NYU-ECNU Institute of Physics at NYU Shanghai, 3663 Zhongshan Road North, Shanghai 200062, China

## Abstract

Proton transfer (PT) processes in solid–liquid phases play central roles throughout chemistry, biology and materials science. Identification of PT routes deep into the realistic catalytic process is experimentally challenging, thus leaving a gap in our understanding. Here we demonstrate an approach using operando nuclear magnetic resonance (NMR) spectroscopy that allows to quantitatively describe the complex species dynamics of generated H_2_/HD gases and liquid intermediates in pmol resolution during photocatalytic hydrogen evolution reaction (HER). In this system, the effective protons for HER are mainly from H_2_O, and CH_3_OH evidently serves as an outstanding sacrificial agent reacting with holes, further supported by our density functional theory calculations. This results rule out controversy about the complicated proton sources for HER. The operando NMR method provides a direct molecular-level insight with the methodology offering exciting possibilities for the quantitative studies of mechanisms of proton-involved catalytic reactions in solid–liquid phases.

## Abstract (web summary)

Identifying proton transfer processes in catalytic reactions is a technically challenging task. Here, the authors employ operando nuclear magnetic resonance spectroscopy to study the reaction mechanism and intermediates of the photocatalytic hydrogen evolution, and to identify the proton sources.

## Graphical abstract

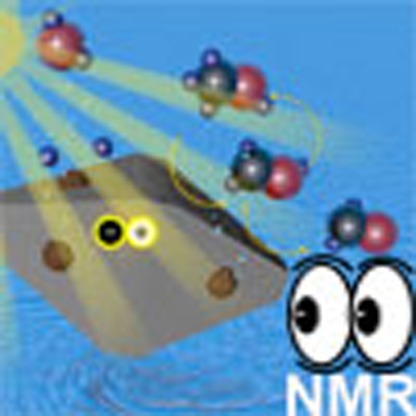

Proton transfer (PT) processes in solid–liquid phases are critically important and can be encountered throughout chemistry, biology and materials science^1^,^2^,^3^,^4^,^5^,^6^,^7^,^8^. The routes and dynamics of PT processes dictate the efficiency of the key biosynthetic and energy conversion systems, both natural and artificial^1^,^2^,^3^,^4^. Hence, tracking the PT trajectories deep into the realistic reaction becomes particularly significant. Molecular hydrogen (H_2_) is a clean-burning fuel that can be produced from protons in the reductive half reaction of solar water splitting where the proton-related species are involved^2^,^9^. Nevertheless, critically important problems including side reactions restrict the productivity of the photocatalytic hydrogen evolution reaction (HER), in which the catalytic steps involve the coupling of light-generated redox equivalents to PT. In these cases, PT in HER is multidirectional. Thus, the addition of specific molecular reagents (also called sacrificial reagents), which is thermodynamically more oxidizable than water (H_2_O), into the reaction environment can suppress the recombination of photogenerated charge carriers and the corresponding reverse reactions to some extent^10^. However, from a fundamental standpoint, some molecular reagents themselves contain protons, such as methanol, ethanol and triethanolamine, which means that such molecular agent itself can be a proton source and this undoubtedly increases the complexity of HER. Therefore, the vague and ambiguous proton source, PT routes, as well as the reaction mechanisms greatly limit our understanding and in turn the development of this field.

Recently, a series of methodologies for mechanism study, such as scanning tunnelling microscopy^11^,^12^,^13^, time-resolved two-photon photoemission^14^, temperature-programmed desorption^15^,^16^,^17^, infrared spectroscopy^18^,^19^,^20^, solid-state nuclear magnetic resonance^21^,^22^,^23^,^24^ and density functional theory calculations (DFT)^25^,^26^ have been used to address the central questions in HER. These methods can simulate and observe a rich surface chemistry of the interaction between adsorbed molecules and catalyst substrates, and distinguish various possible HER mechanisms. However, few of these techniques alone permit the direct quantification of specific species in reactions, and, moreover, the vast majority of them usually operate under ideal conditions (solid–gas or in ultrahigh vacuum), albeit with exquisite resolution, and it is still a quite tough task to take into account the complexities of practical conditions such as solvent effect, surface specificity and adsorbate–adsorbate interactions. For instance, the adsorption and dissociation of methanol (CH_3_OH) gas can occur easily on anatase-TiO_2_ (101) surface under continuous ultraviolet illumination; however, in the liquid solution weak interactions such as hydrogen-bonding effects between CH_3_OH and H_2_O may significantly complicate the reaction and probably impair the conclusion/mechanism derived from the ideal reaction conditions. Furthermore, some possible hydrolysates in HER are out of ability to be observed under the gas–solid vacuum condition, and some changes that occur during the reaction may no longer be apparent when the sample returns to a non-working state. In this regard, there still exists a large gap between over-idealized conclusions and realistic heterogeneous reaction systems. Yet, to the best of our knowledge, no experimental evidence for quantitative insight into the PT mechanisms of HER in solid–liquid phases has been reported.

Here we demonstrate an approach, using operando NMR spectroscopy, that allows to directly quantify the populations of H_2_/HD gases and liquid intermediates in pmol resolution, as well as the tracking of the PT routes deep into the realistic heterogeneous HER conditions, which contains Pd/TiO_2_ (anatase), H_2_O and CH_3_OH. In this system, the effective protons for HER are mainly from H_2_O, and CH_3_OH evidently serves as an outstanding sacrificial agent reacting with holes. Furthermore, it also provides evidence to rule out controversy about the complicated sources of protons for HER and the role of methanol as sacrificial molecules, with the methodology offering possibilities for the quantitative mechanism studies of proton-related catalytic reactions.

## Results

### Experimental set-up of operando NMR

**Figure 1:**
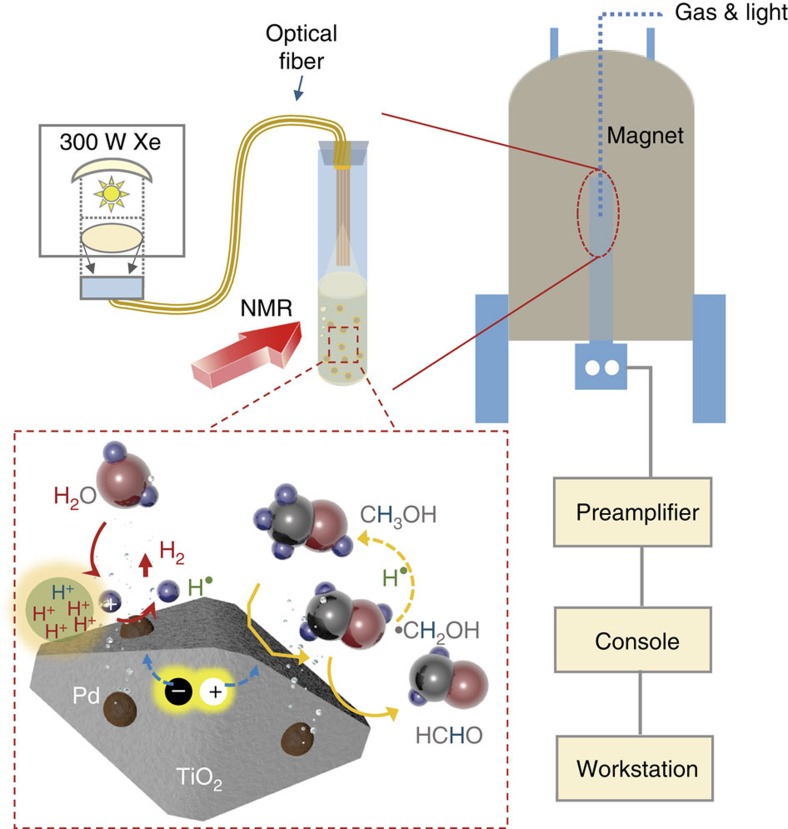
Schematic layout of set-up for operando NMR studies. The set-up consists of a micro-reactor system based on a homemade NMR tube, allowing solid–liquid heterogeneous mixture to be directly studied inside the NMR coil while maintaining good uniformity. The light source (300-W Xe lamp) beam was directed through a focusing lens assembly to converge on the entrance face of a homemade optical fibre bundle (seven quartz fibres, diameter 3 mm).

Our experimental set-up consists of a micro-reactor system based on a NMR tube, allowing solid–liquid heterogeneous mixture to be directly studied inside the NMR coil. The experimental scheme is illustrated in Fig. 1 and Supplementary Fig. 1. The light source (300 W Xe lamp) beam is directed through a focusing lens assembly to converge on the entrance face of a homemade optical fibre bundle (seven quartz fibres, diameter 3 mm). The NMR study is incident on a suspension containing solid photocatalyst together with the aqueous mixtures, and the signals originate from not only the solid photocatalyst itself but also from the different gas–liquid molecules and chemical species dissolved in the solution^27^,^28^. This designed scheme has provided excellent feature information among the solid, liquid and gas mixtures without the separation of products. Our NMR studies are carried out on 700-MHz Agilent NMR spectrometers at a magnetic field strength of 16.4 T. An Agilent 5 mm z-axis pulsed field gradient triple resonance probe (^1^H {^13^C, ^15^N}) is used (Detailed information can be found in Supplementary Fig. 2 and Supplementary Note 1).

To operando probe molecular species qualitatively and quantitatively in the solid–liquid phases, we measured some benchmark experiments (Supplementary Figs 3–10). First, the ^1^H NMR signals of aqueous suspensions containing TiO_2_, CH_3_OH and D_2_O were examined (Supplementary Figs 7 and 8). Two resonance peaks were clearly observed, corresponding to the methyl group (CH_3_-) of CH_3_OH (Supplementary Fig. 8b) and the residual protons of D_2_O (Supplementary Fig. 8a)^29^. Interestingly, the signal of H_2_ (singlet, 4.57 p.p.m.) was also remarkably detected in the spectrum of the suspension when the H_2_ gas was injected into it (Supplementary Fig. 9). This could provide the way to quantitatively evaluate the amount of H_2_ in the aqueous solution by comparing with the signal from the solution of saturated dissolved H_2_. Second, the signal sensitivity was evaluated. Take 3-(trimethylsilyl)-1-propanesulfonic acid sodium salt (DSS) for example; when we decreased the content of DSS to nearly 50.7 pmol, the signal still had a relatively good signal-to-noise ratio (Supplementary Fig. 10). Even though by unitary sampling (number of scans is 1) of the heterogeneous mixture, the signals still had a good signal-to-noise ratio and thus could be distinctly distinguished. Therefore, the signals measured by the operando NMR could have pmol sensitivity, indicating a good capability to operando probe the gas–liquid intermediate products quantitatively deep into the solid–liquid working conditions at the very early stage of the reaction.

### Operando NMR spectroscopy measurements

In a photocatalysis system, electrons are shuttled to the reductive side where they reduce protons to H_2_. However, because of the complexity in building and optimizing such a complete system, when studying the reductive half reaction, it is common for a sacrificial electron donor to be used to optimize catalysts for H_2_ production. Thus, for a clear recognition of the PT routes, it is significant to clarify the roles of the sacrificial agents. Widely accepted explanations of the roles for sacrificial agents (for example, methanol) are the thermodynamically more oxidizable ability than water, which work as an external driving force for the surface chemical reactions^12^. Their effects have been attributed to the consumption of holes, with the aim of suppressing the recombination of photogenerated charge carriers and the surface back reaction, which results in an increased H_2_ evolution^30^. Yet, no experimental evidence for this mechanism was reported. On the other hand, some groups (for example, Xu *et al.*^15^ and Highfield *et al.*^19^) pointed out that photo-reforming of methanol (gas) over the photocatalyst may also produce pure H_2_, which makes it plausible to assume that the protons from methanol sacrificial agent have participated in the HER. However, these experimental simulations under ideal conditions depend on assumptions and simplifications, and many questions concerning details of the mechanisms in real conditions without the separation of products remain unanswered. As a consequence, the vague and ambiguous roles of sacrificial agents greatly limit our understanding of the PT routes.

**Figure 2:**
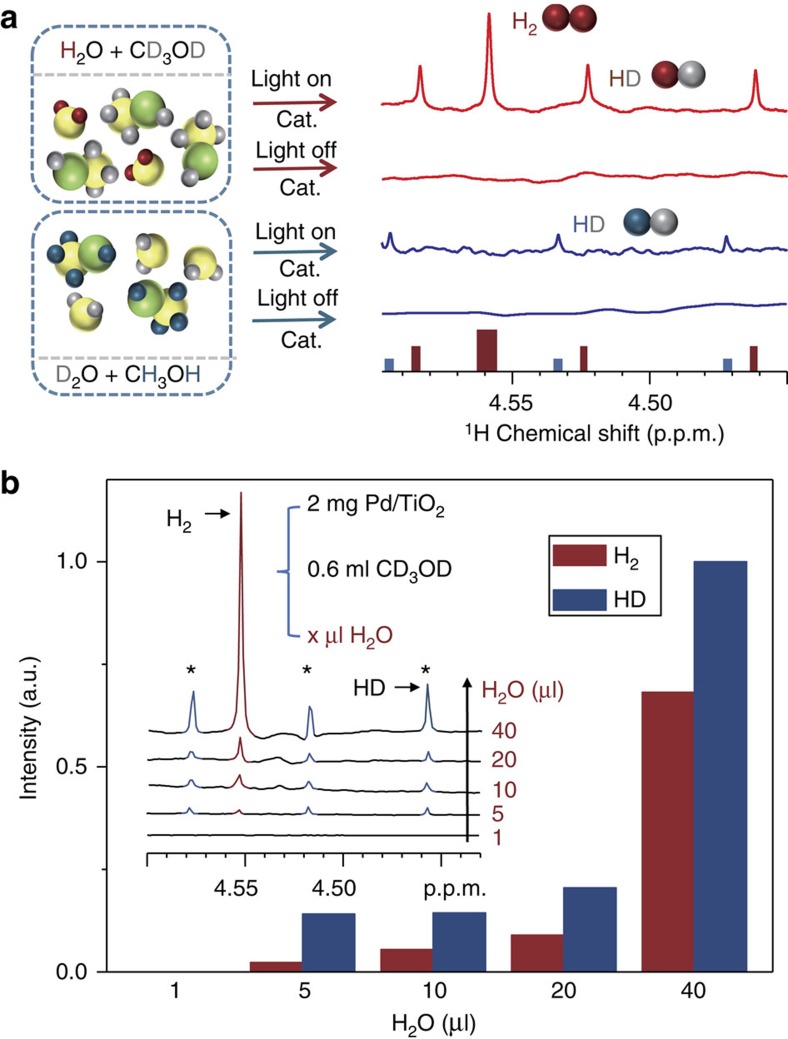
^1^H NMR spectra of different systems. (**a**) ^1^H NMR spectra of H_2_/HD gases produced from heterogeneous systems before and after the irradiation with UV/Vis light. The spectra were acquired from samples containing (A,B) CH_3_OH (20 μl), D_2_O (500 μl) and Pd/TiO_2_ (2 mg) at 298 K with a 5 s recycle delay and 16 scans before (A) and after (B) the UV/Vis irradiation for 24 h; (C, D) H_2_O (20 μl), CD_3_OD (500 μl) and Pd/TiO_2_ (2 mg) at 298 K with a 5 s recycle delay and 32 scans before (C) and after (D) the UV/Vis irradiation (300-W Xe lamp) for 2 h. The shifts of the H_2_/HD peaks are caused by the different deuterated reagents. (**b**) ^1^H NMR spectra of the correlations between produced H_2_/HD gas and added H_2_O. The NMR experiments were acquired from samples containing Pd/TiO_2_ (2 mg), CD_3_OD (600 μl), D_2_O (1% DSS as the internal reference) and H_2_O (*x* μl, *x*=1, 5, 10, 20, 40) at 298 K with a 5 s recycle delay and 32 scans after the UV/Vis irradiation for 2 h.

To clarify these issues, NMR was applied on two deliberately designed reaction systems, namely System 1 (H_2_O/CD_3_OD/catalyst) and System 2 (CH_3_OH/D_2_O/catalyst), to provide some qualitative preliminary results. The virtue of those systems lays on the variation of the protonated agent of the similar reaction, which allows selective monitoring of the PT routes in HER. Figure 2a shows ^1^H NMR spectra of these two systems before and after the irradiation with ultraviolet/visible (UV/Vis) light. No H_2_/HD NMR signal is observed for these samples in the dark. Upon irradiation for 2 h, the H_2_ signal (single, 4.56 p.p.m.) and the HD signal (triplet, 4.46, 4.52 and 4.57 p.p.m.) resonances are clearly observed for System 1. Whereas only trace amount of HD gas (triplet, 4.47, 4.53 and 4.58 p.p.m.) is found for System 2, although this system has been irradiated for 24 h before the NMR study. Considering the solution compositions of System 1, we conclude that the proton source of the H_2_ gas in the photocatalysis system, H_2_O/CH_3_OH/catalyst, is at least partial from H_2_O definitely.

For a deeper understanding, we changed the concentration of H_2_O of System 1. As can be seen from Fig. 2b and Supplementary Fig. 11, it is observed that the signal intensity of H_2_ gas in the NMR spectra increases with the content of the adding H_2_O. This is another direct evidence for its involvement in PT (from H_2_O to H_2_), which can support our deduction for the origin of H_2_ in System 1. Note that the H/D isotope effect has been realized in literature^31^,^32^. The presence of the H/D isotope effect can decrease the reaction rate of water splitting; however, in principle will not harm our deduction for the origin of H_2_ in the studied reaction system.

The origin of HD gas is an intriguing question and also related to the photocatalytic mechanism. In a mixture of H_2_O and CD_3_OD, few HDO and CD_3_OH species can be generated because of the H/D exchange reactions^33^, which further increases the complexity of the origin of HD gas. For System 1, there are two possibilities for the origin of HD gas: the product of HDO or the reforming product of CD_3_OH. As inspired by the work of Yang *et al.*^17^, the methyl group H of CH_3_OH would transfer to the O_BBO_ sites of TiO_2_ (anatase) when it is photocatalytically dissociated, while proton reduction reaction prefers to occur on the surface of cocatalyst rather than bulk TiO_2_. Consistent with this interpretation, a control experiment without loaded Pd cocatalyst cannot generate any H_2_/HD product (Supplementary Fig. 13). Thus, the origin of HD gas is likely from HDO. In order to verify our hypothesis, some compared experiments for the system of D_2_O/CH_3_OH/TiO_2_ were carried out (Supplementary Fig. 14). The volume ratios selected for CH_3_OH are from 5 to 20%. In these samples, few HDO and CH_3_OD species would be generated. After the irradiation with UV/Vis light for 7 h, no H_2_/HD NMR signal is observed. Thus, we can safely infer that the reduction process for CH_3_OH/CH_3_OD to produce H_2_/HD is quite limited within 7 h in this system. That is to say, the origin of HD gas in System 1 is mainly from HDO. Similarly, in System 2, we prolonged the illumination time to 24 h; only trace amount of HD gas was found (this might indicate that the concentration of HDO is much more smaller than D_2_O in this sample). The results provide a compelling evidence that the dominating proton source of H_2_ gas in the HER of CH_3_OH/H_2_O/catalyst mixture (CH_3_OH Vol %<20%) is H_2_O species, and H_2_ gas from the photo-reforming process of CH_3_OH in the CH_3_OH/H_2_O mixture solution is negligible.

Note that in a mixture of H_2_O and CD_3_OD, some H_3_O^+^ and CH_3_OH_2_^+^ (as well as OH^−^ and CH_3_O^−^) species may also be generated by autoprotolysis. In this study, the pH value of the system is 7. The influence of the concentration of H_3_O^+^/OH^−^ and CH_3_OH_2_^+^/CH_3_O^−^ on the photocatalytic reaction is the ongoing study in our laboratory.

**Figure 3:**
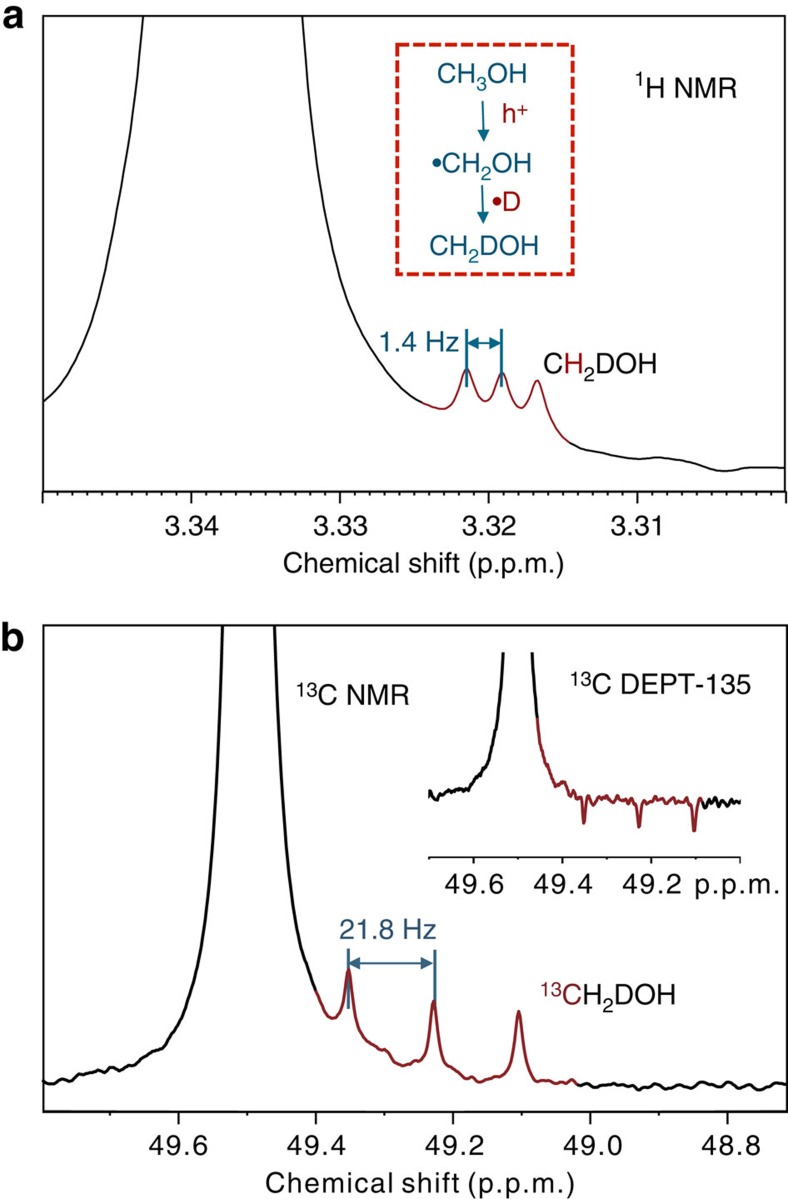
High resolution NMR spectra of as-prepared samples. (**a**) ^1^H NMR spectrum of the methanol intermediates after the UV/Vis irradiation. The ^1^H NMR experiment was acquired from samples containing Pd/TiO_2_ (2 mg), CH_3_OH (20 μl) and D_2_O (500 μl) at 298 K with a 5 s recycle delay and 16 scans after the UV/Vis irradiation for 40 h. (**b**) ^13^C NMR spectrum of the methanol intermediates after the UV/Vis irradiation. The ^13^C NMR experiment was acquired from samples containing Pd/TiO_2_ (2 mg), ^13^CH_3_OH (20 μl) and D_2_O (500 μl) at 298 K with a 2 s recycle delay and 32 scans after the UV/Vis irradiation for 40 h. Inset is the DEPT-135 NMR spectrum of the as-prepared samples. The DEPT-135 spectrum was acquired from samples containing Pd/TiO_2_ (2 mg), ^13^CH_3_OH (20 μl) and D_2_O (500 μl) at 298 K with a 1 s recycle delay and 32 scans after the UV/Vis irradiation for 40 h. In the DEPT-135 spectrum, the negative ^13^C signals indicate that the signals are from the partially deuterated methyl (−CH_2_D).

To get a better understanding of the role of methanol, a series of ^13^C-labelled methanol (^13^CH_3_OH) instead of the naturally abundant component was added in the reaction system (D_2_O/^13^CH_3_OH/catalyst). Figure 3 shows the ^1^H/^13^C NMR spectra of methanol intermediates of the reaction system containing Pd/TiO_2_, D_2_O and CH_3_OH. In this system, CH_3_OD would be generated because of the fast H/D exchange reactions. Interestingly, after irradiating for 40 h, the ^1^H NMR spectrum shows a well-resolved triplet signal with equal intensities (1:1:1 triplet, *J*=1.4 Hz) at the right side of the CH_3_ peak of CH_3_OH(D) (Fig. 3a). While similar triplet signals can also be observed from the ^13^C NMR spectrum (Fig. 3b). According to the chemical shift and the characteristic *J* coupling value, we have assigned these two signals to CH_2_D- of CH_2_DOH(D). The possibility of -CHD- can be easily ruled out by the observation in the 1D ^13^C DEPT-135 (Distortionless Enhancement by Polarizition Transfer) spectrum where the triplet signals show clear negative intensities (Fig. 3b, inset).

For the formation of CH_2_DOH(D), two processes can be proposed: one is through an addition reaction of HCHO and HD, while the other is through the reaction of ·CH_2_OH(D) with D radicals (·D). It has been reported that the average adsorption energy of HCHO is much lower than that of CH_3_OH; therefore, it may desorb from the TiO_2_ surface quickly rather than recombine with an H/D atom^19^. Furthermore, as will be discussed in a further publication, the HCHO may transform into methanediol species quickly in the aqueous solution, which accelerates the desorption rate of HCHO. It is therefore reasonable to conclude that the CH_2_DOH(D) is likely formed through a coupling reaction of ·CH_2_OH(D) and ·D, providing an indirect evidence of the existence of ·CH_2_OH(D) radicals during the HER. Meanwhile, ·CH_2_OH(D) is the inevitable intermediate when CH_3_OH(D) reacts with a hole (h^+^), which gives a definitive evidence that CH_3_OH serves as a sacrificial agent and plays a crucial role in mediating the capture of holes to form ·CH_2_OH radicals. To our knowledge, ·CH_2_OH species only exists in the liquid (may not be present in the ideal conditions) and is hard to be captured when it is in an isolated state, while it would also rapidly convert to HCHO (as also confirmed by DFT), thus resulting in a lacking experimental evidence and unconfirmed mechanisms for this important process.

### DFT studies

**Figure 4:**
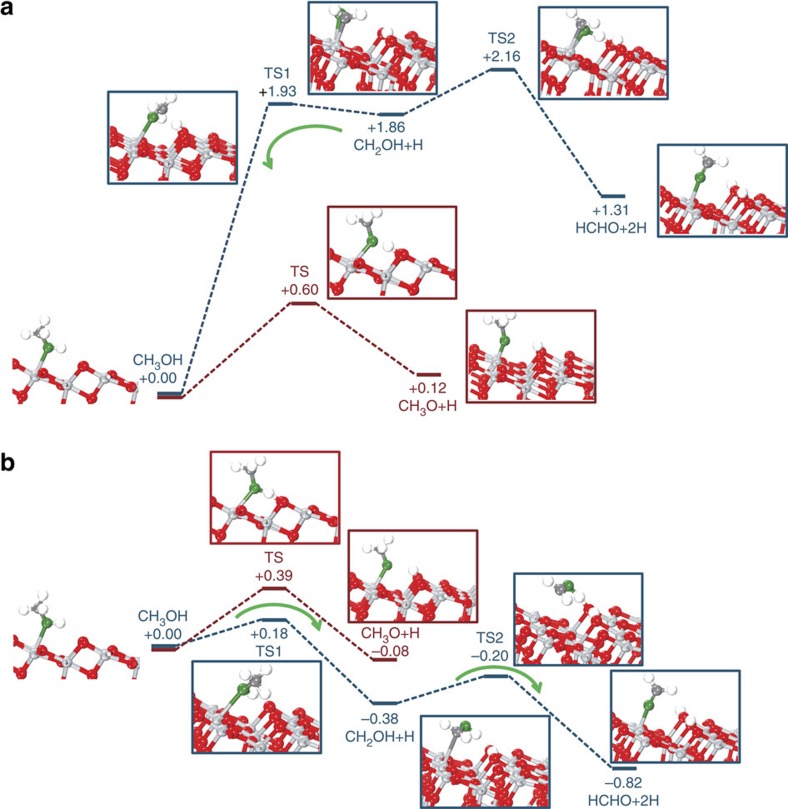
DFT calculations. (**a**) DFT of the dissociation of CH_3_OH at the ground state. (**b**) Photo-oxidation of CH_3_OH begins with C–H broken (blue line) and O–H broken (red line). The calculated structures are displayed in the profile. The Ti atoms are in light grey and O in red, while the C atoms are in dark grey and H in white, and O atom of CH_3_OH are in green. This notation is used throughout this paper.

For a better understanding of the reaction mechanism, we have also conducted systematic DFT calculations concerning the reactions of CH_3_OH at both ground and excited states on the anatase-TiO_2_ (101) surface^34^,^35^. The results are illustrated in Fig. 4a (ground state) and Fig. 4b (excited state). As one can see, at the ground state, the dissociation of methanol through breaking of O–H or C–H bond is obviously an endothermic process. Therefore, the CH_3_OH may stay intact at the surface under dark conditions. Interestingly, with the help of h^+^, both C–H and O–H bond-breaking reactions become exothermic, and the C–H dissociation is more favourable both thermodynamically and dynamically compared with the O–H dissociation. It indicates that CH_3_OH may prefer to react with h^+^ to form ·CH_2_OH species under excited conditions. Then, ·CH_2_OH species may react with ·H (·D) back to methanol or further react with h^+^ to HCHO. Both reactions have also been studied in our calculations, and it turns out that, at the ground state, the ·CH_2_OH may readily convert back to methanol with the barrier of only 0.07 eV—much lower than the further oxidation to HCHO (0.30 eV). By contrast, at the excited state, CH_2_OH prefers the further oxidation to HCHO with a barrier of 0.18 eV rather than returning to methanol, which has a much higher barrier of 0.56 eV.

### Quantitative operando NMR spectra

Operando NMR can not only provide qualitative insight into the catalytic mechanism but also be fully quantitative deep into the real solid–liquid phases. In our system, H_2_ gas is a very important product, and its variation tendency may reflect some important microcosmic catalytic processes. Although some other characterization techniques such as gas chromatography (GC) and mass spectrometer (MS) can also be used to measure the gas products, they can only detect the gases that escape from the solution (after the dissolved gas reaches the saturation concentration), whereas in fact quite amount of gas molecules might be already present in the solution with partial dissolution and adsorption at the early stage of the reaction. Furthermore, the microcosmic reaction processes and trends before the dissolved gas reaches the saturation concentration will be missing, and the processes could last for more than 10 min for the 40 nmol H_2_ dissolving in 100 ml H_2_O solution. Meanwhile, it is also impossible for GC or MS to probe some intermediates that cannot escape from the reaction solution. In this context, the operando NMR approach is superior to GC or MS in studying the HER.

**Figure 5:**
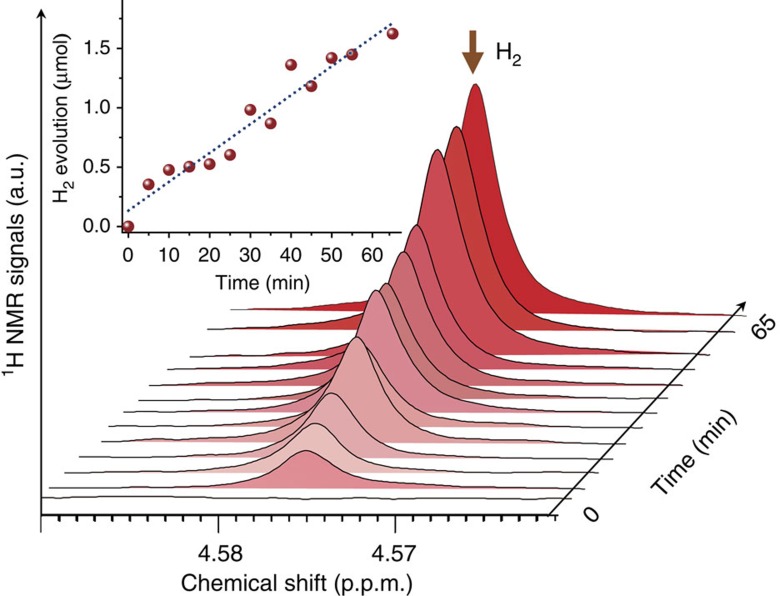
Quantitative operando ^1^H NMR spectra. Quantitative time-related ^1^H NMR spectra of Pd/TiO_2_ at 298 K in H_2_O/CD_3_OD mixtures under UV/Vis light irradiation. The operando NMR experiments were acquired from samples containing Pd/TiO_2_ (1 mg), CD_3_OD (300 μl), D_2_O (1% DSS as the internal reference) and H_2_O (300 μl) at 298 K with a 5 s recycle delay and eight scans under the UV/Vis irradiation. The first 1,000 points of the FID was cut off to obtain the final separable H_2_ signals.

In our system, assuming that the H_2_ gas does not reach the saturation state at the early stage of the reaction, the observed H_2_ signals in the NMR spectra could reflect the whole H_2_ gas from HER. H_2_ gas can be quantified by using the intensity of the H_2_ signal in the H_2_–D_2_O saturation solution (estimate 0.018 ml H_2_ per 1 ml D_2_O) at the same temperature (Supplementary Figs 15 and 16). Through comparison of the H_2_ resonance intensities with calibration samples, it is possible to quantify the number of H_2_ species in absolute terms at the early stage and gain insight into the PT behaviour properties in working state. Figure 5 shows the quantitative operando ^1^H NMR spectrum of H_2_O/CD_3_OD/catalyst system. For a suitable comparison with the results obtained from the conventional GC detector (Supplementary Fig. 12), the sample was irradiated directly by an Xe light source. The H_2_ signal intensity increased linearly on continuous illumination. Under this condition, no HD signal can be detected, and this can be because of the few exchanged HDO products compared with H_2_O, consistent with the previous results. The quantification result in Fig. 5 shows that the H_2_ evolution rate at the early stage from the NMR measurement is ∼1.560 μmol h^−1^ mg^−1^. The above result may be far-reaching because it indicates that the widely used methods have the intrinsic shortcoming on the detection of the reaction especially at the early stage. As a consequence, the reaction models derived from the *ex situ* measurement, especially the models for the reaction kinetics, will deviate from the actual reaction mechanisms. Thus, this operando NMR spectroscopic method provides an approach to guide the design of HER systems, offering better measures deep into the realistic reaction.

## Discussion

By using an operando NMR method, we successfully quantified the generation of H_2_/HD gases and liquid intermediates in pmol resolution without the separation of products, and provided evidence for the PT routes during the HER. On the basis of the photocatalytic system containing anatase-TiO_2_, methanol and water, we find that the effective protons for HER are mainly from water, and methanol evidently serves as an outstanding electronic sacrificial agent reacting with holes. However, for the sacrifice reforming process of methanol, a small amount of released methyl group protons still occur, which are likely bound to the surface of TiO_2_, and their migration to cocatalyst to take part in the HER would consume more time and energy. This indicates that complicated synergistic effect between water and methanol exists under practical HER conditions. The picture in Supplementary Fig. 17 illustrates the paramount PT processes together with the reaction mechanism of HER. Carrier generation and recombination occur when an electron transfers from the valence band to conduction band in a semiconductor. Once the photoexcited charges reach the surface of the particle, they can take part in surface redox reactions with adsorbed donor or acceptor molecules^34^,^35^,^36^,^37^. In our system, the induced electron transfers from the TiO_2_ conduction band to the metal particles, and the attached proton would be reduced by an electron to its monoatomic radical species (·H), which is the main predecessor of H_2_ (Supplementary Fig. 17, (equation 5)). On the other side, the holes would be consumed by CH_3_OH, extracting the charges competitively with their recombination and then improving the formation rate of H_2_. On the oxidative side, two sequential hole consumptions convert the adjacent CH_3_OH species to ·CH_2_OH (equation 2) and HCHO (equation 3), respectively, accompanying with the release of protons. Subsequent spontaneous (dark) coupling reaction between ·CH_2_OH and ·H radicals results in CH_3_OH species re-formation (equation 4). On the reductive side, protons would be reduced to hydrogen by the shuttled electrons on the surface of cocatalyst (equation 5). In H_2_O/CH_3_OH (CH_3_OH vol % <20%) mixtures, H_2_O molecules would provide a vast amount of free protons in solution (equation 1). Although the photocatalytically dissociated protons from CH_3_OH are constrained by the O_BBO_ nearby, only a slim chance for those to be reduced to H_2_ exists. As a consequence, the current NMR spectroscopic studies provide a unique viewing angle for us to understand the predominant PT processes of HER in solution.

The results in this work deepen our understanding of interfacial heterogeneous redox reactions, and the operando NMR method will be applicable extensively with the methodology offering exciting possibilities for the microcosmic mechanism study (pmol resolution) in catalysis, photoelectrocatalysis and electrocatalysis of reactions such as CO_2_ reduction, waste-water remediation and methanol reforming.

## Methods

### Synthesis of catalyst

The Pd(1.0 wt %)/TiO_2_ photocatalyst powder was prepared by the co-precipitation method. Briefly, appropriate amount of PdCl_2_ aqueous solution (1 wt%) was added into the TiO_2_ (100 mg, Anatase, Sigma) powder and maintained at 80 °C for 1 h. After being dried, the products were calcined at 300 °C for 2 h. Before being characterized and tested, the samples were reduced in 20% H_2_/Ar at 300 °C for 1 h.

### NMR experiment

All NMR experiments were acquired on 700 MHz Agilent NMR spectrometers at a magnetic field strength of 16.4 T. An Agilent 5 mm z-axis pulsed field gradient triple-resonance static probe was used in all ^1^H and ^13^C NMR experiments. All ^1^H NMR measurements were carried out at 298 K with a spectral width of 20 p.p.m., pulse width of 4 μs (45^o^), a recycle delay of 5 s and 8, 16 or 32 scans. To suppress the water signal, the first 1,000 points of the free induction decay (FID) were cut off before FT. ^13^C NMR was carried out at 298 K with a spectral width of 253 p.p.m., pulse width of 7.3 μs (45^o^), a recycle delay of 2 s and 32 scans. All experiments had DSS as the internal reference. To enhance the NMR signals, the examples could also be directly irradiated by an Xe light source during the irradiation accumulation process.

*Operando*^*1*^*H NMR studies*. A 300-W Xe lamp was used as the light source. The light source is outside the magnet. The light source beam was directed through a focusing lens assembly to converge on the entrance face of a homemade optical fibre bundle (seven quartz fibres, diameter 3 mm). The cap of the tube was specially designed to allow the input of the light and the H_2_ gas. Note that the fibre may lead to an attenuation of the light intensity and a lower reaction rate (see Fig. 5 and Supplementary Fig. 18). In order to simulate the HER process similar to the conventional GC, the samples might be irradiated directly by an Xe light source.

### Calculation details

All the calculations were carried out using the Vienna *ab initio* simulation package^38^,^39^, and the exchange-correlation term was described by the Perdew, Burke and Ernzerhof version within the generalized gradient approximation^40^. The project-augmented wave^41^,^42^ method was used to represent the core–valence electron interaction. The titanium 3*s*, 3*p*, 3*d*, 4*s* and the carbon and oxygen 2*s*, 2*p* electrons were treated as valence electrons and an energy cutoff of 400 eV for the basis-set expansion was used.

The anatase-TiO_2_ (101) surface was modelled by a five trilayer slab with only the centre layer fixed, and other atoms were allowed to relax until atomic forces reached below 0.05 eV Å^−1^. As suggested by Luo and co-workers^43^, the hole was introduced by using the triplet state to mimic the singlet excited state^43^,^44^,^45^,^46^,^47^. A 3 × 1 surface cell and a >15 Å vacuum gap was used. Different k-point meshes were tested, and it was found the k-point sampling restricted to the Γ point only can already provide reliable results regarding adsorption energies.

The transition states in reactions were located with a constrained optimization scheme^48^, and were verified when (i) all forces on atoms vanish and (ii) the when total energy is maximum along the reaction coordinate but minimum with respect to the rest of the degrees of freedom.

### Data availability

All relevant data are available from the authors.

## Additional information

**How to cite this article:** Wang, X. L. *et al.* Operando NMR spectroscopic analysis of proton transfer in heterogeneous photocatalytic reactions. *Nat. Commun.* 7:11918 doi: 10.1038/ncomms11918 (2016).

## Supplementary Material

Supplementary Information Supplementary Figures 1-18 and Supplementary Note 1
