# Supplementary material for: Operando NMR spectroscopic analysis of proton transfer in heterogeneous photocatalytic reactions
Source: Nat Commun. 2016 Jun 17;7:11918. doi: 10.1038/ncomms11918 (PMC4915033; doi:10.1038/ncomms11918)
Supplement: Supplementary Information — Supplementary Figures 1-18 and Supplementary Note 1 [file ncomms11918-s1.pdf]

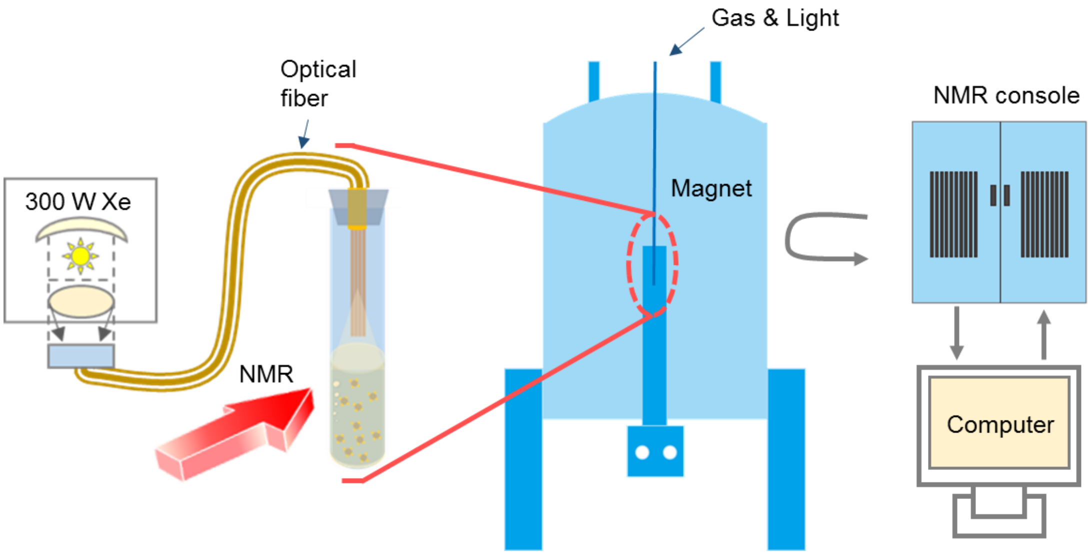

Supplementary Figure 1. Schematic layout of set-up for operando NMR studies.

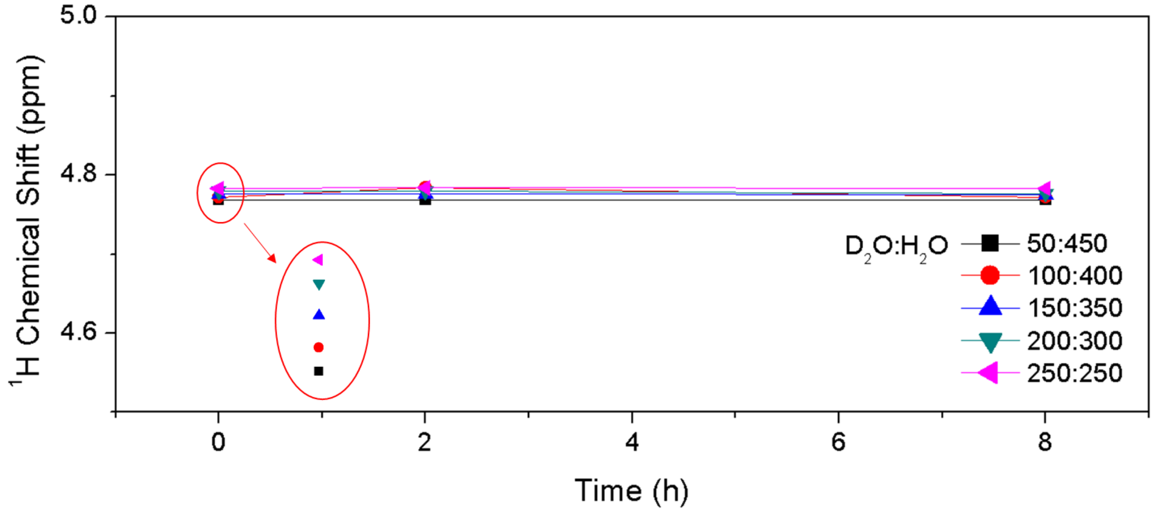

Supplementary Figure 2. Correlations between different ratios of D_2O/H_2O and ^1H chemical shifts of HDO. The spectra were acquired from samples containing different ratios of D_2O and H_2O (D_2O:H_2O 50 µL:450 µL, 100 µL:400 µL, 150 µL:350 µL, 200 µL:300 µL, 250 µL:250 µL) at 298 K with a 5 s recycle delay and 32 scans.

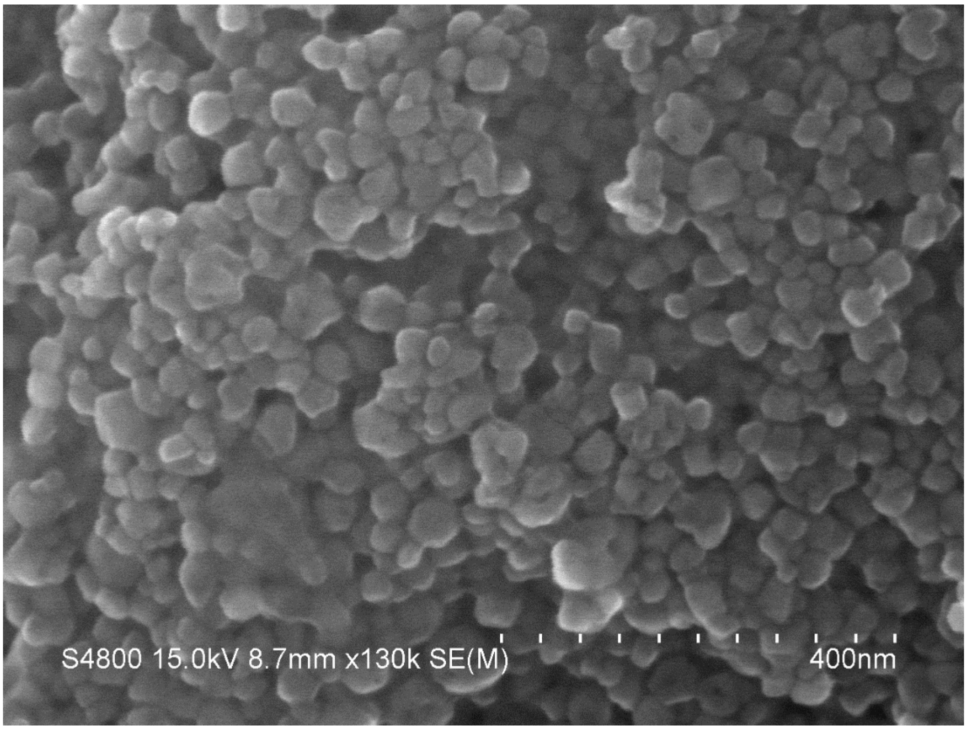

Supplementary Figure 3. SEM image of as-prepared anatase-TiO_2 nanoparticles. The diameter of as-prepared anatase-TiO_2 sample is ranging from 20 to 40 nm.

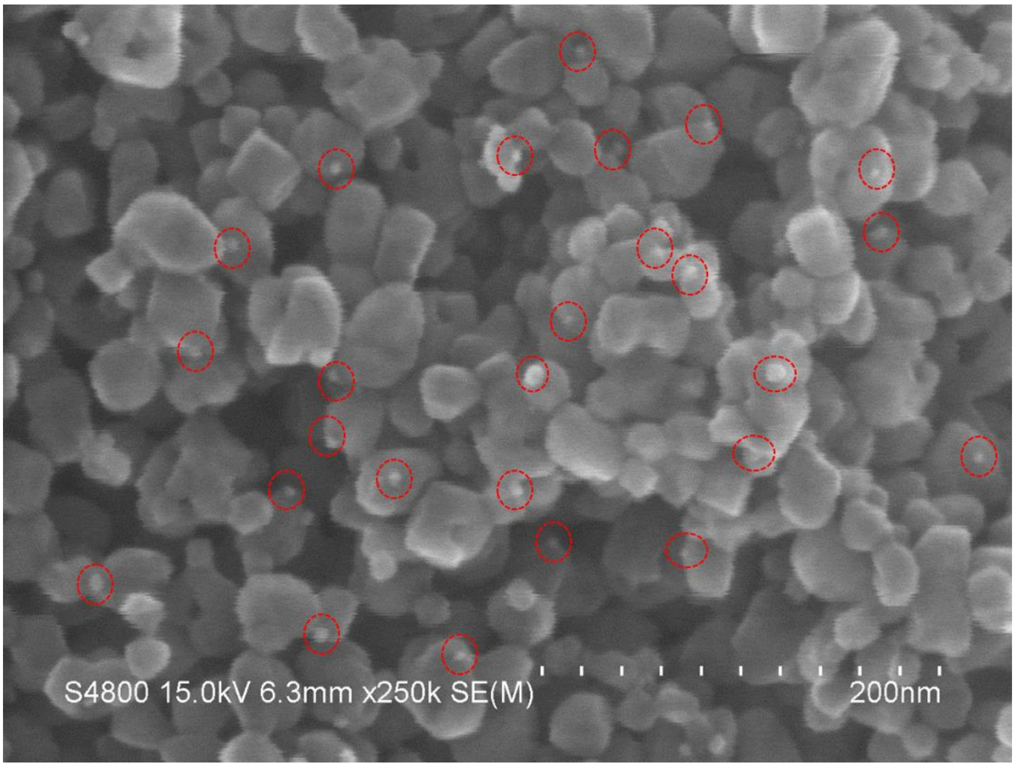

Supplementary Figure 4. SEM image of Pd/TiO_2 sample. The Pd(1.0 wt %)/TiO_2 photocatalyst powder was prepared by the co-precipitation method. Briefly, appropriate amount of $PdCl_2$ aqueous solution (1 wt%) was added into the TiO_2 (100 mg, Anatase, Sigma) powder and maintained at 80 ^oC for 1 h. After being dried, the products were calcined at 300 ^oC for 2 h. Prior to being characterized and tested, the samples were reduced in 20 % H_2/Ar at 300 ^oC for 1 h. Pd particles loaded on TiO_2 were marked by red dotted line circles.

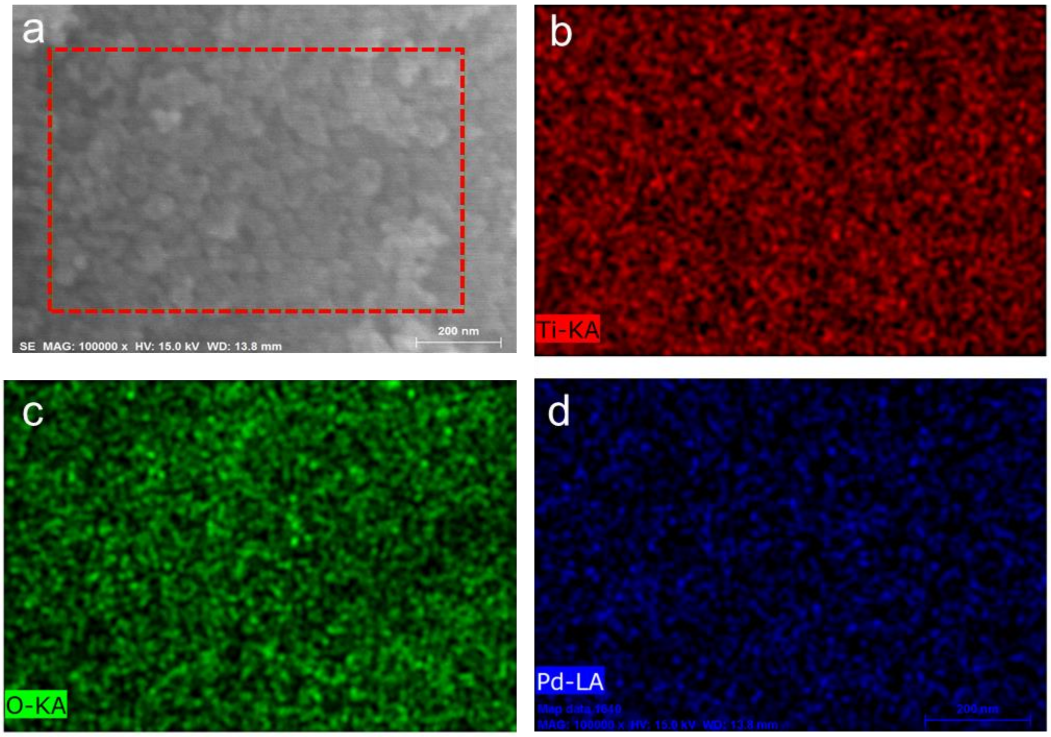

Supplementary Figure 5. SEM-EDS elemental mapping of Pd/TiO_2 sample. (**a**) SEM image of as-synthesized Pd/TiO_2 sample; (**b**), (**c**) and (d) EDS elemental mapping of Ti (red), O (green) and Pd (blue), respectively. The EDS mapping analysis implies that Pd particles are uniformly distributed on the surface of TiO_2 photocatalyst.

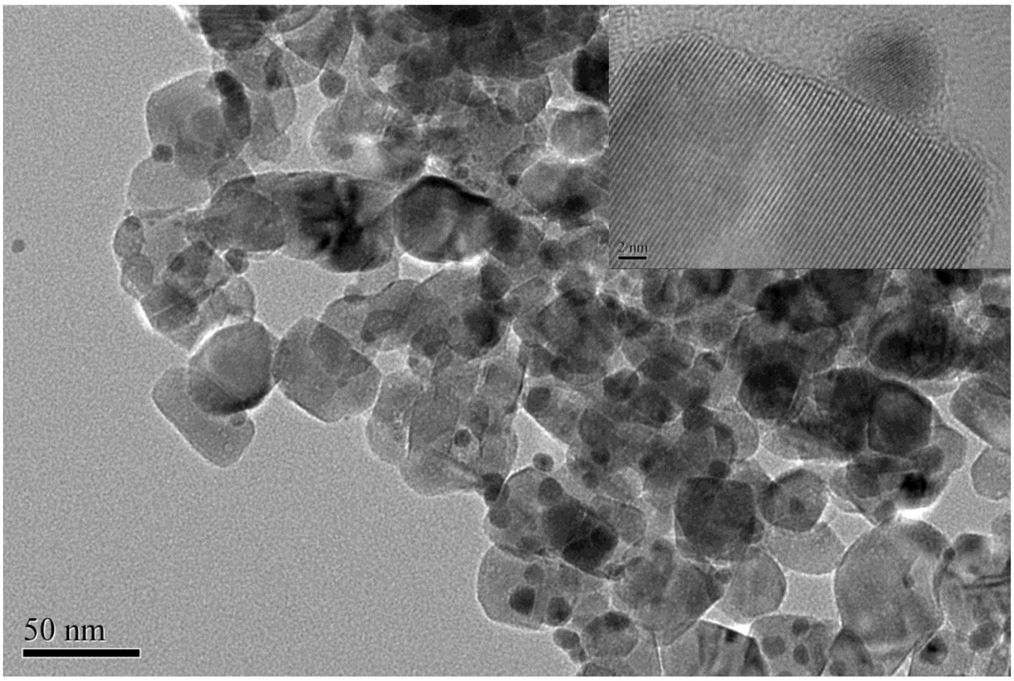

Supplementary Figure 6. TEM image of Pd/TiO_2 sample.

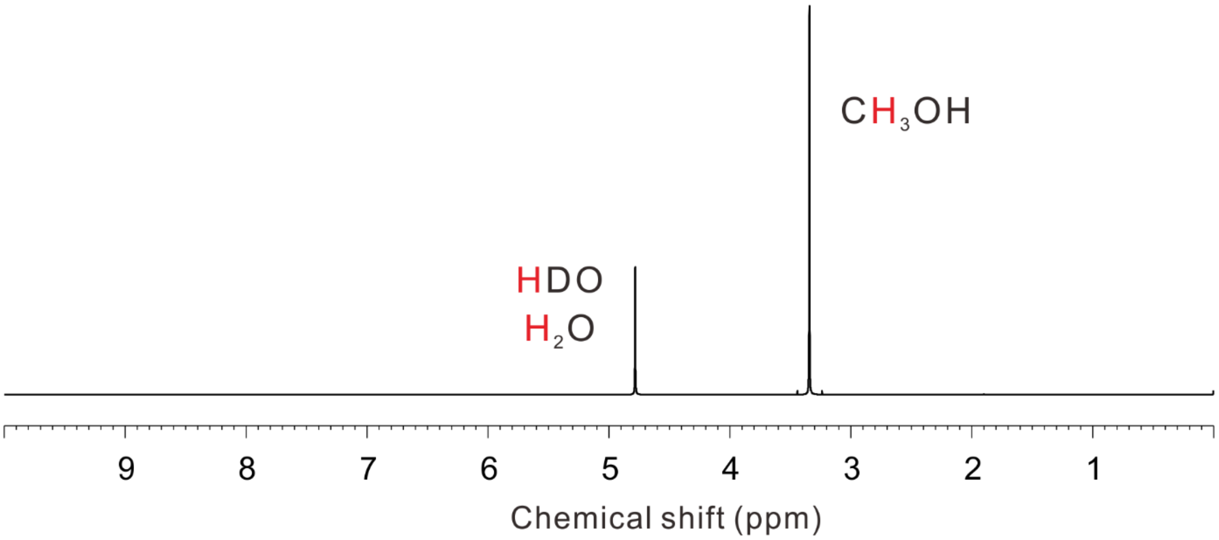

Supplementary Figure 7. ^{1}H NMR spectrum of the as-prepared sample. The spectrum was acquired from sample containing TiO_2 (2 mg), D_2O (500 μL) and CH_3OH (20 μL) at 298 K with a 5 s recycle delay and 16 scans in the dark. Two resonance peaks were clearly observed at 3.35 and 4.78 ppm, corresponding to methyl group (CH_3-) of CH_3OH and the residual protons of D_2O, respectively.

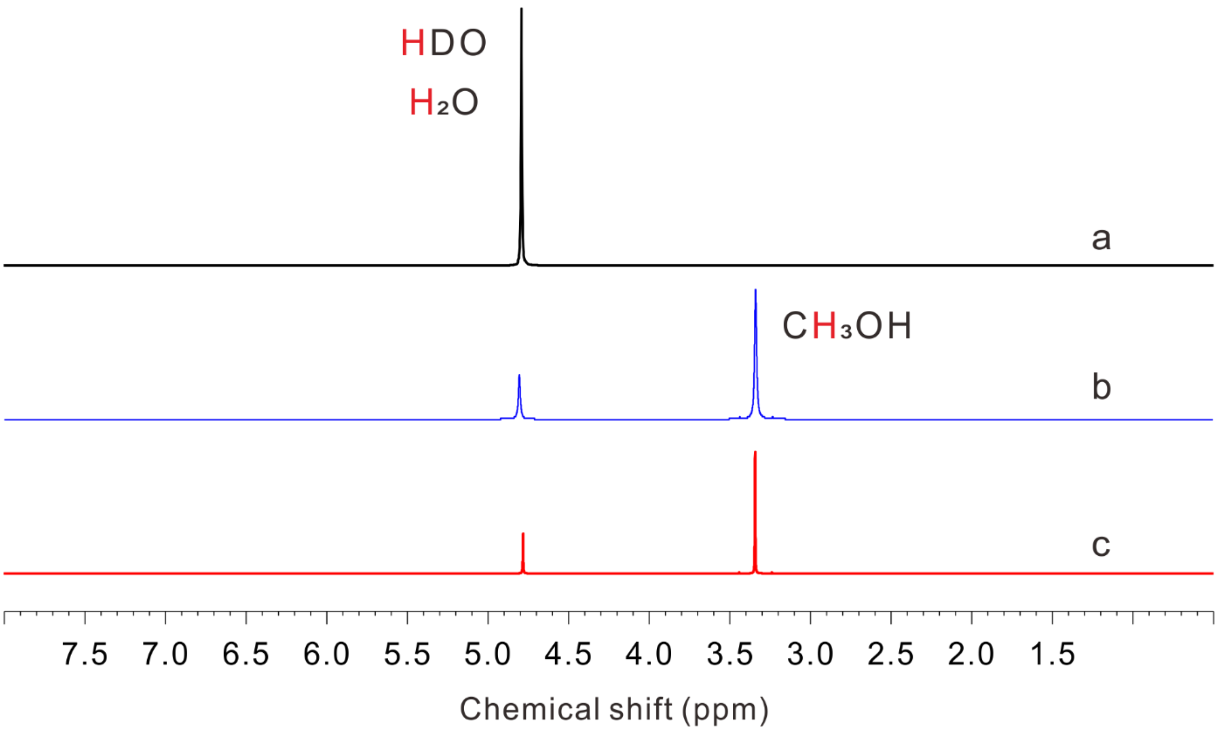

Supplementary Figure 8. ^{1}H NMR spectra of the as-prepared samples. ^{1}H NMR spectra of sample containing (**a**) 500 μL D_2O, (**b**) 500 μL D_2O and 20 μL CH_3OH, and (**c**) 2 mg TiO_2, 500 μL D_2O and 20 μL CH_3OH suspensions at 298 K with a 5 s recycle delay and 16 scans. It is noted that TiO_2 particles were uniformly dispersed in the solution and contributed no signal.

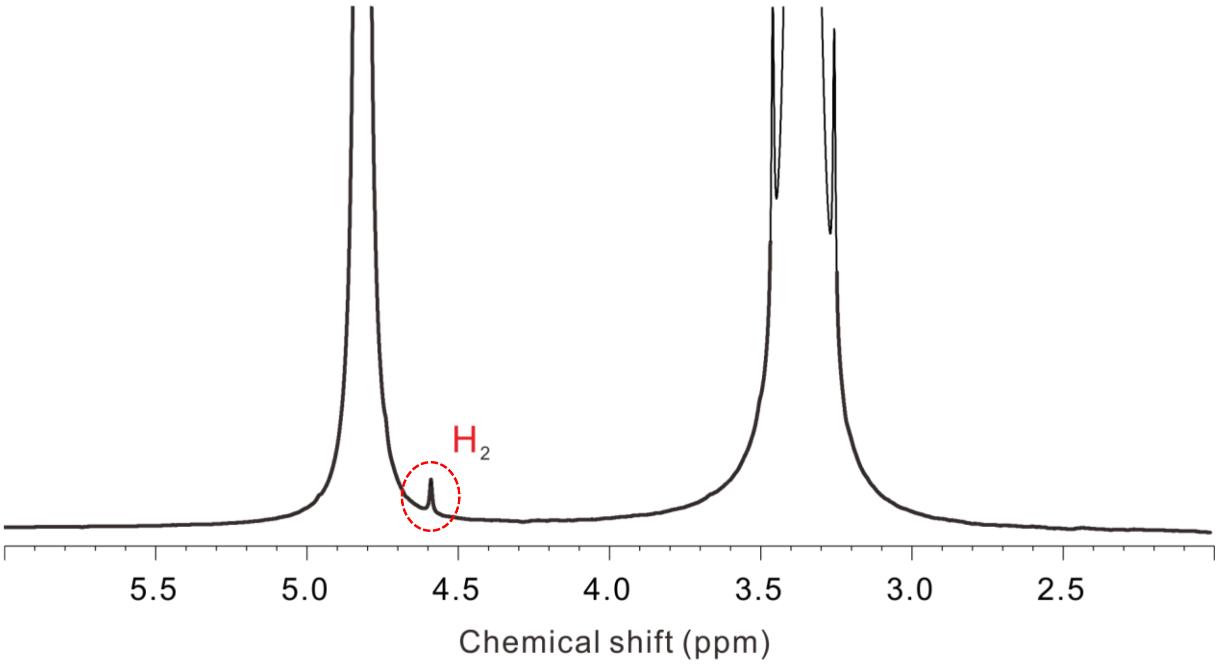

Supplementary Figure 9. ^{1}H NMR spectrum of the suspensions containing gas, solid and liquid. The spectrum was acquired from samples containing TiO_2 (2 mg), D_2O (500 μL) and CH_3OH (20 μL), and then H_2 was injected under the pressure of 3.5 bar. The as-prepared sample needed to stand for 5 min before measurement. Interestingly, the signal of H_2 (singlet, 4.57 ppm) was remarkably detected in the spectrum of the suspension when the H_2 was injected into it.

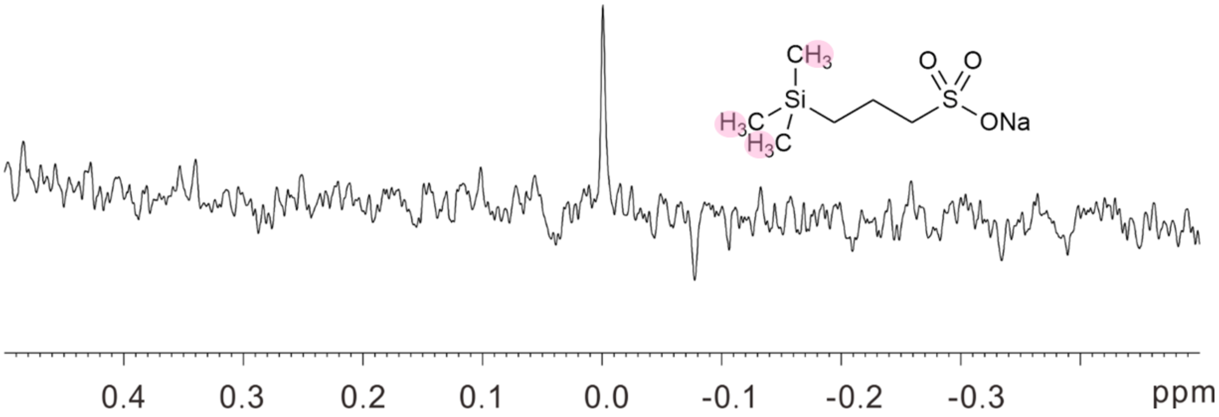

Supplementary Figure 10. ^{1}H NMR spectra of the DSS ($(CH_3)_3Si(CH_2)_3SO_3Na$) sample. The high resolution ^{1}H spectra were acquired on 700 MHz equipment from sample containing D_2O (500 μL) and D_2O (contains 10^{-3} % (w/w) DSS, 1μL) at 298 K with 5 s recycle delay and 32 scans.

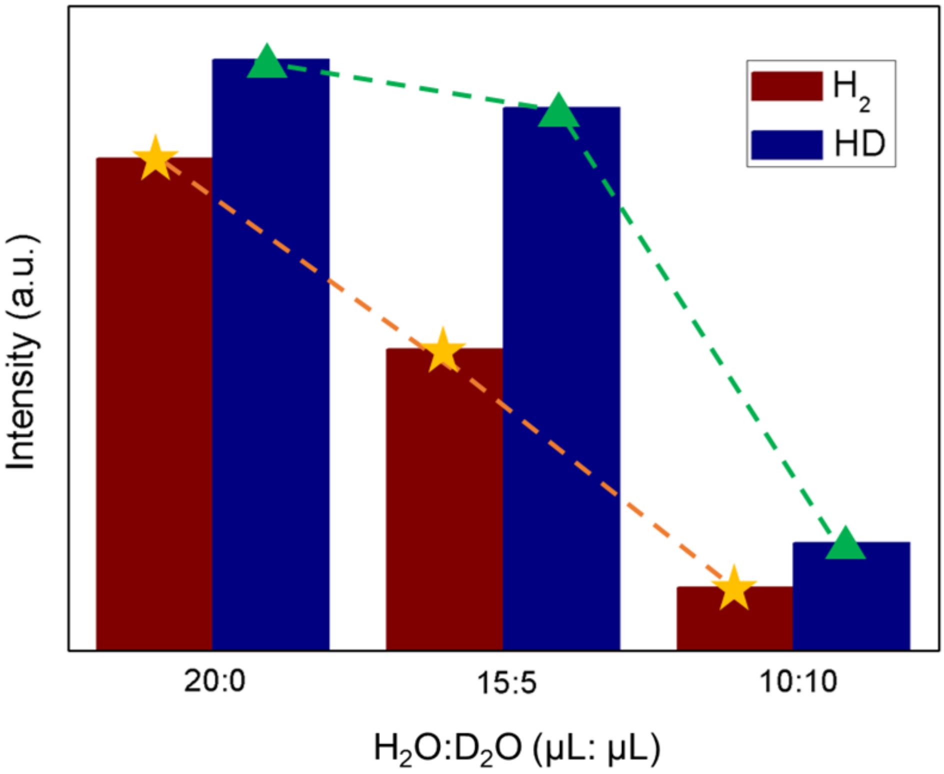

Supplementary Figure 11. ^{1}H NMR signal intensity of the prepared samples. ^{1}H NMR signal intensity of H_2/HD gases produced from heterogeneous systems containing Pd/TiO_2 (2 mg), CD_3OD (500 μL), H_2O and D_2O (20 μL:0 μL, 15 μL:5 μL and 10 μL:10 μL) at 293 K with a 5 s recycle delay and 32 scans after the UV/Vis irradiation (300 W Xe lamp) for 4 h.

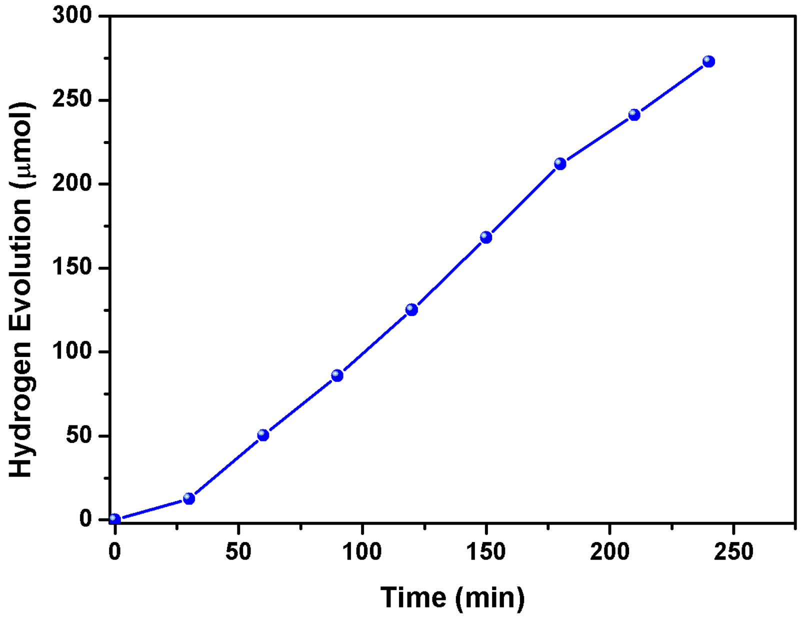

Supplementary Figure 12. Photocatalytic H_2 evolution of Pd/TiO_2 under UV-visible light irradiation. The photocatalytic reaction was performed in a pyrex glass cell connected to a glass closed gas circulation system. H_2 evolution analysis was performed by dispersing 200 mg of catalyst power in an aqueous solution containing methanol (100 mL, 10 vol.%) as the sacrificial electron donor. The reactant solution was evacuated several times to remove air completely before the reaction. A 300 W Xe lamp was used as the light source. H_2 evolution, starting as soon as the UV/Vis light was on, increased linearly during the irradiation.

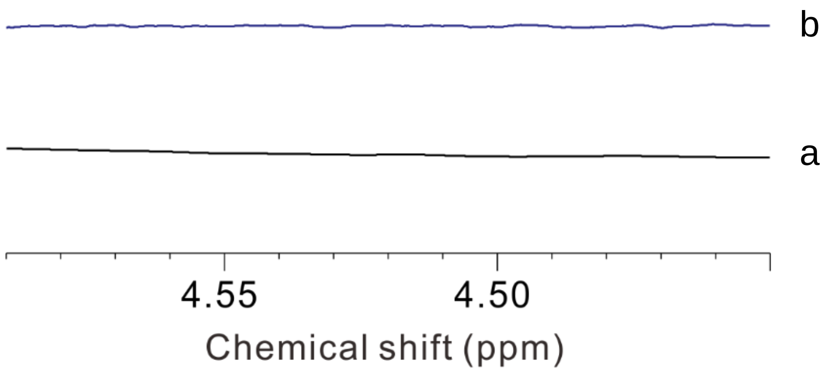

Supplementary Figure 13. ^{1}H NMR spectra of the control experiment samples without loaded Pd cocatalyst. ^{1}H NMR spectra of the sample before (a) and after (b) the irradiation. The high resolution ^{1}H spectra were acquired on 700 MHz equipment from sample containing TiO_2 (2 mg), D_2O (500 μL) and CH_3OH (20 μL) at 298 K with 5 s recycle delay and 16 scans before (a) and after (b) the UV/Vis irradiation (300 W Xe lamp, $\lambda > 300$ nm) for 24 h. The control experiments have shown that the H_2 and HD gases did not be acquired at all in the dark or in the absence of Pd species, confirming that the reduction process is a photocatalytic process and the Pd species behave as the active sites for the hydrogen evolution reaction.

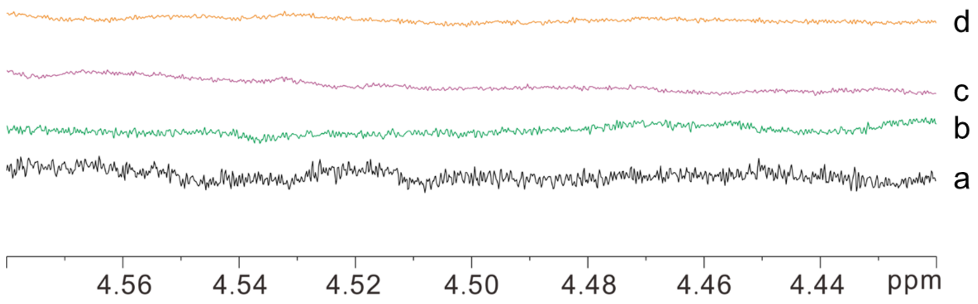

Supplementary Figure 14. ^{1}H NMR spectra of the sample after the UV irradiation. The high resolution ^{1}H spectra were acquired on 700 MHz equipment from sample containing Pd/TiO_2 (2 mg), D_2O and CH_3OH (D_2O:CH_3OH 475 μL:25 μL, 450 μL:50 μL, 425 μL:75 μL and 400 μL:100 μL) at 298 K with 5 s recycle delay and 32 scans after the direct UV/Vis irradiation (300 W Xe lamp, $\lambda > 300$ nm) for 7 h.

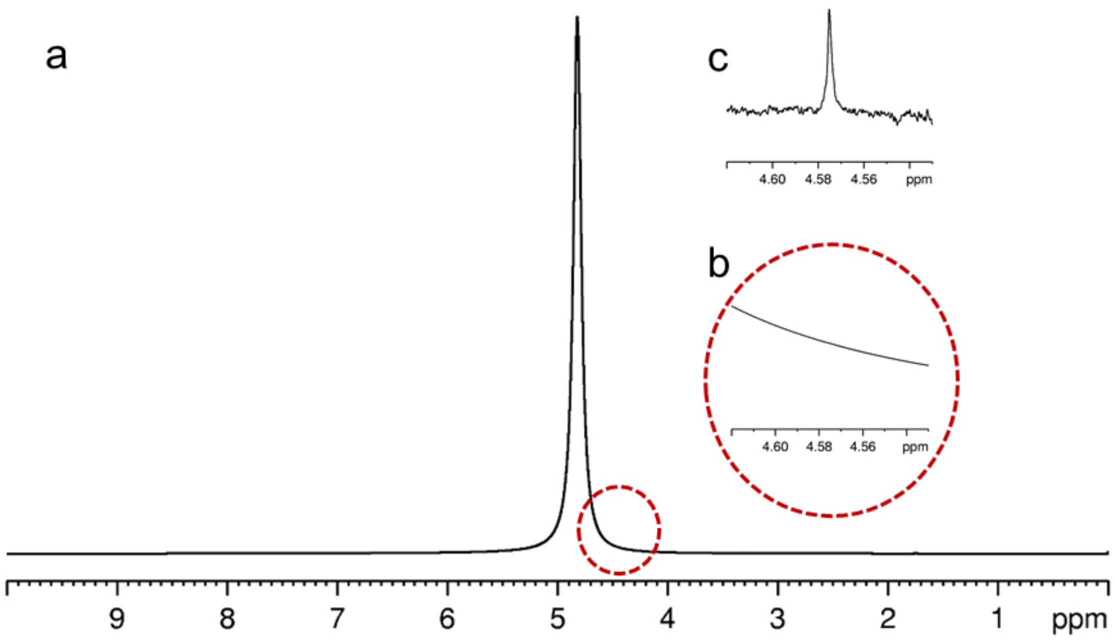

Supplementary Figure 15. ^{1}H NMR spectra of the as-prepared samples after the irradiation. (**a**, **b**) The high resolution ^{1}H spectra were acquired on 700 MHz equipment from sample containing TiO_2 (2 mg), H_2O (500 μL) and CD_3OD (100 μL) at 298K with 5 s recycle delay and 8 scans under the UV/Vis irradiation by fiber (300 W Xe lamp, $\lambda > 300$ nm). (**c**) The first 1000 points of the FID has been cut off.

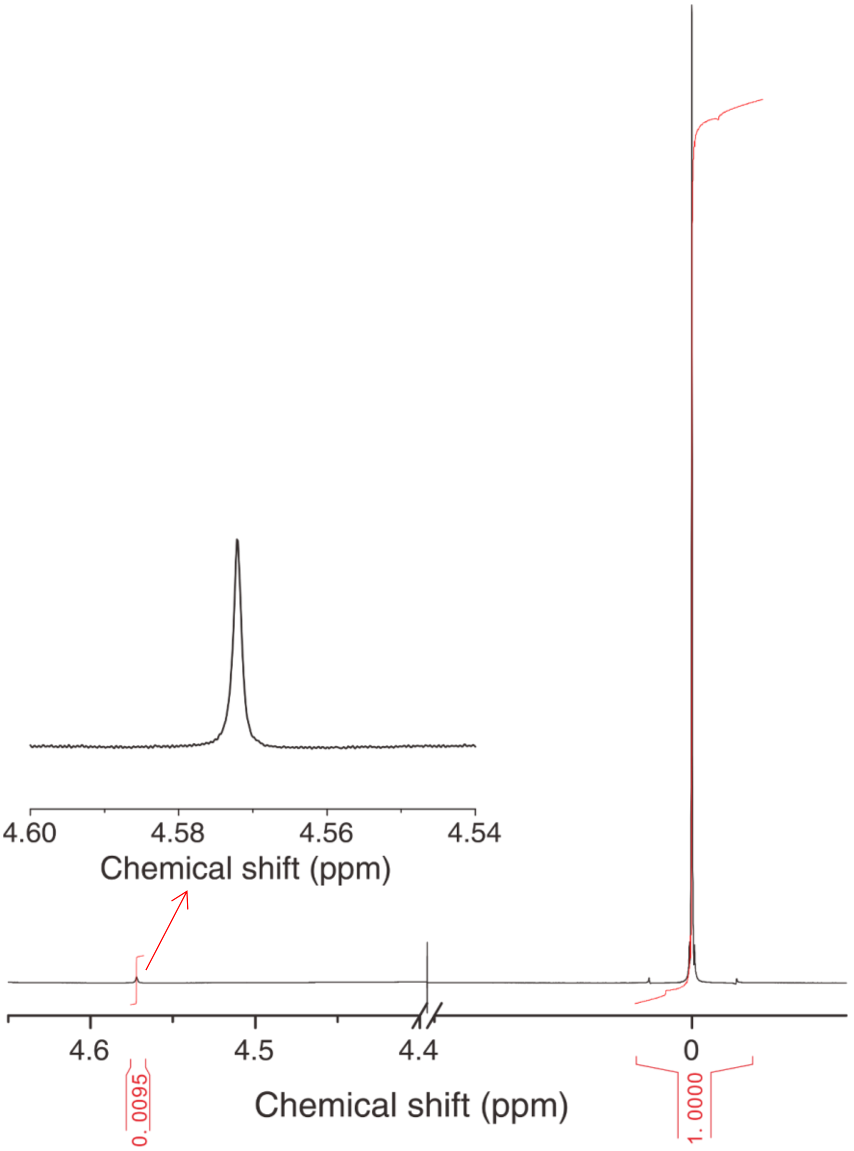

Supplementary Figure 16. ^1H NMR spectra of the saturated H_2. The high resolution ^1H spectra were acquired on 700 MHz equipment from sample containing D_2O (500 μL) and saturated H_2 at 298K with 5 s recycle delay and 32 scans. H_2 was injected into the D_2O with the pressure maintaining at 3.5 bar. Before investigating, the samples needed to stand for 5 min. Through comparison of the H_2 resonance intensities with saturated sample, it is possible to quantify the number of H_2 species in absolute terms.

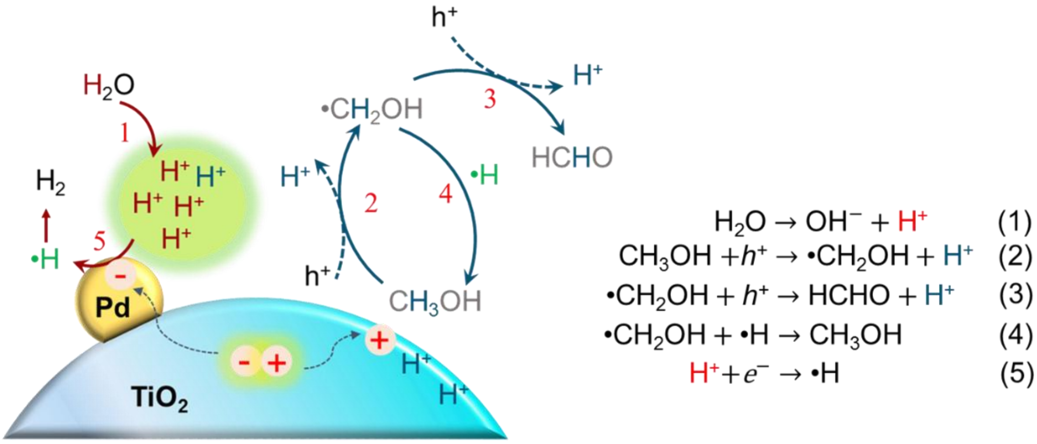

$$H_2O \rightarrow OH^- + H^+ \quad (1)$$

$$CH_3OH + h^+ \rightarrow \bullet CH_2OH + H^+ \quad (2)$$

$$\bullet CH_2OH + h^+ \rightarrow HCHO + H^+ \quad (3)$$

$$\bullet CH_2OH + \bullet H \rightarrow CH_3OH \quad (4)$$

$$H^+ + e^- \rightarrow \bullet H \quad (5)$$

Supplementary Figure 17. Proposed mechanism for UV-light driven water reduction using methanol as sacrificial electron donator. After illumination, the electron-hole pair is subsequently separated, and then the electron transfers from the TiO_2 conduction band to the Pd particles, and the attached H^+ is reduced to •H, which is the main predecessors of H_2. On the other side, methanol serves as the electronic sacrificial agent, and the reforming route can be clearly seen from the equations on the right.

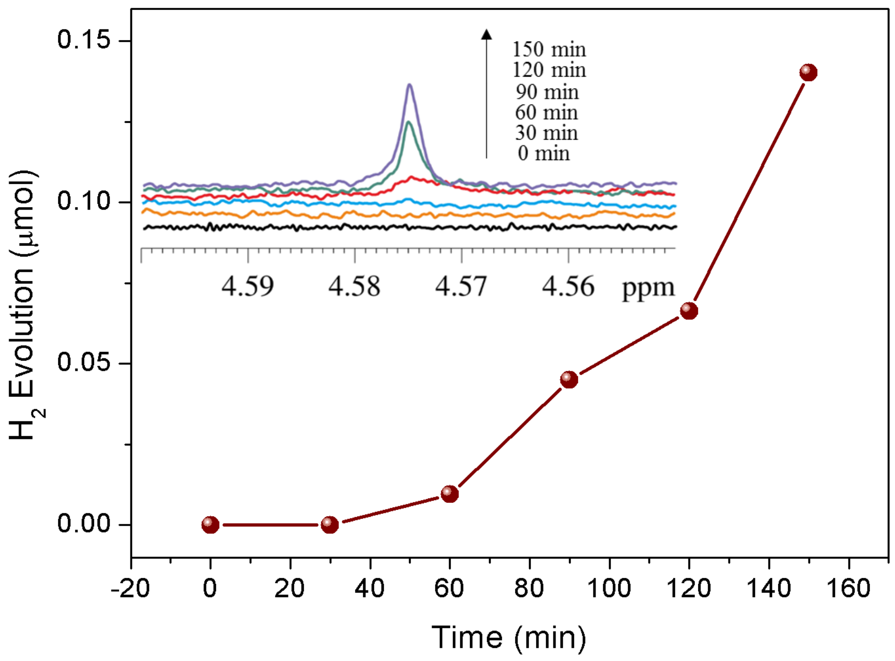

Supplementary Figure 18. Quantitative operando ^{1}H NMR spectra. Quantitative time-related ^{1}H NMR spectra of Pd/TiO_2 at 298 K in H_2O/CD_3OD mixture under UV-visible light irradiation through the man-made fiber. The operando NMR experiments were acquired from samples containing Pd/TiO_2 (1 mg), CD_3OD (50 μL), D_2O (1 % DSS as the internal reference) and H_2O (500 μL) at 298 K with a 5 s recycle delay and 32 scans under the UV/Vis irradiation. The first 1000 points of the FID was cut off to obtain the final separable H_2 signals.

Supplementary Note 1: An NMR spectrometer consists of a superconducting magnet, a probe, a radio transmitter, a radio receiver, an analog-to-digital converter (ADC), and a computer (Fig. 1 and Supplementary Fig. 1). The computer directs the transmitter to send a high-power and very short duration pulse of radio frequency to the probe coil. Immediately after the pulse, the weak signal (FID) received by the probe coil is amplified, converted to an audio frequency signal, and sampled regular intervals of time by the ADC to produce a digital FID signal. The computer determines the timing and intensity of pulses output by the transmitter and receivers and processes the digital information supplied by the ADC. After the computer performs the Fourier transform, the resulting spectrum can be displayed on the computer monitor and plotted on paper with a digital plotter.

Before carrying out our experiments, some phenomena need to be taken into account. First, we consider magnetic field effects on this photocatalytic reaction. Kiwi et al. have reported that H· spin remains unaffected when increasing the field strength and no increase or decrease in the rate of recombination of paramagnetic species such as H· involved in the catalytic process has taken place. As a consequence, the magnetic field interaction with the TiO_2 related catalysts might not lead to any variation in the final H_2. Just in case, we remained the magnetic field strength unchanged (16.4 T) during the experiments.

Second, the kinetics of H/D exchange were measured systematically. Note that in a mixture of ^1H and $D(^2H)$ reagents, few H/D species can be generated due to the H/D exchange reactions. Hence, we tested the kinetics of H/D exchange. Take H_2O and D_2O for examples,

different ratios of H_2O:D_2O (50 μmol:450 μmol, 100 μmol:400μmol, 150 μmol:350 μmol, 200 μmol:300 μmol and 250 μmol:250 μmol) mixtures were transferred into the standard NMR tube (5 mm). The “settling time” (the time interval between the preparation of the H_2O:D_2O mixtures and NMR test) is about 5 min. As can be seen from Supplementary Fig. 2, no obvious ^{1}H NMR shift of HDO (H/D exchange product from H_2O and D_2O) peaks can be detected until 8 h. As a result, we can safely deduce that the H/D exchange reaction would reach a stable during the settling time. Considering the optical intensity, some other improvements are ongoing in our laboratory.
